# A novel mouse model of *PMS2* founder mutation that causes mismatch repair defect due to aberrant splicing

**DOI:** 10.1038/s41419-021-04130-8

**Published:** 2021-09-06

**Authors:** Kajal Biswas, Martin Couillard, Luca Cavallone, Sandra Burkett, Stacey Stauffer, Betty K. Martin, Eileen Southon, Susan Reid, Teri M. Plona, Ryan N. Baugher, Stephanie D. Mellott, Kristen M. Pike, Mary E. Albaugh, Chelsea Maedler-Kron, Nancy Hamel, Lino Tessarollo, Victoria Marcus, William D. Foulkes, Shyam K. Sharan

**Affiliations:** 1 Mouse Cancer Genetics Program, Center for Cancer Research, National Cancer Institute, National Institutes of Health, Frederick, MD USA; 2 The Lady Davis Institute of the Jewish General Hospital, McGill University, Montreal, QC Canada; 3 Leidos Biomedical Research, Inc. Frederick National Laboratory for Cancer Research, Frederick, MD 21702 USA; 4 CLIA Molecular Diagnostics Laboratory, Leidos Biomedical Research, Inc. Frederick National Laboratory for Cancer Research, Frederick, MD USA; 5 Department of Pathology, McGill University, Montreal, QC Canada; 6 Department of Oncology, McGill University, Montreal, QC Canada; 7 Department of Human Genetics, McGill University, Montreal, QC Canada; 8 Department of Medical Genetics, Jewish General Hospital, McGill University, Montreal, QC H3T 1E2 Canada; 9 Department of Medical Genetics and Cancer Research Program, Research Institute of the McGill University Health Centre, McGill University, Montreal, QC H4A 3JI Canada

**Keywords:** Cancer genetics, Colon cancer

## Abstract

Hereditary non-polyposis colorectal cancer, now known as Lynch syndrome (LS) is one of the most common cancer predisposition syndromes and is caused by germline pathogenic variants (GPVs) in DNA mismatch repair (MMR) genes. A common founder GPV in *PMS2* in the Canadian Inuit population, NM_000535.5: c.2002A>G, leads to a benign missense (p.I668V) but also acts as a de novo splice site that creates a 5 bp deletion resulting in a truncated protein (p.I668*). Individuals homozygous for this GPV are predisposed to atypical constitutional MMR deficiency with a delayed onset of first primary malignancy. We have generated mice with an equivalent germline mutation (*Pms2c.1993A>G*) and demonstrate that it results in a splicing defect similar to those observed in humans. Homozygous mutant mice are viable like the *Pms2* null mice. However, unlike the *Pms2* null mice, these mutant mice are fertile, like humans homozygous for this variant. Furthermore, these mice exhibit a significant increase in microsatellite instability and intestinal adenomas on an *Apc* mutant background. Rectification of the splicing defect in human and murine fibroblasts using antisense morpholinos suggests that this novel mouse model can be valuable in evaluating the efficacy aimed at targeting the splicing defect in *PMS2* that is highly prevalent among the Canadian Inuits.

## Introduction

Pathogenic variants in the DNA mismatch repair (MMR) genes such as, *MLH1, MSH2, PMS2*, and *MSH6* are associated with various human malignancies [1, 2]. Bi-allelic GPVs in MMR genes leads to a severe phenotype, constitutional MMR deficiency (CMMRD), that is characterized by early onset of leukemia/lymphoma, colorectal/gastrointestinal tumors, brain tumors, and rhabdomyosarcoma [3]. Mono-allelic GPVs in MMR genes predisposes individuals to hereditary non-polyposis colorectal cancer (HNPCC), better known now as Lynch syndrome (LS) [4]. The mismatch repair (MMR) protein PMS2 (Post-meiotic segregation increased 2) interacts with another MMR protein MLH1 (MutL Homolog 1) and the MLH1-PMS2 heterodimer plays a central role in post-replicative DNA mismatch repair [5]. While mutations in mismatch repair genes *MLH1* and *MSH2* are more prevalent (42–50% for MLH1 and 33–39% for MSH2), *PMS2* mutations are less frequent (<7%) in families with LS [4]. The low penetrance of *PMS2* in cancer is possibly due to the presence of homolog protein MLH3 which is able to bind MLH1 and partially function in MMR [6, 7]. Tumors with deficiency in MMR genes display a molecular phenotype characterized by genomic instability of several microsatellite repeats present throughout the genome and is referred to as microsatellite instability (MSI) [8].

A number of unique mutations have been identified in various MMR genes, including several variants of uncertain clinical significance that are listed in InSiGHT database (https://www.insight-group.org/variants/databases/). One of the most frequently occurring mutations in *PMS2* is a founder GPV found in the Inuit population of Nunavik, Quebec, Canada [9]. This GPV in exon 11 of *PMS2* (NM_000535.5:c.2002A>G, (p.I668V)) generates a de novo splice site that competes with the authentic site, resulting in aberrant splicing and consequently deletion of five bases from exon 11 at the exon 11–12 junction in the mRNA and is predicted to generate a truncated protein [9]. Interestingly, although the variant is limited to Nunavik and the western coastline of Hudson Bay, it is predicted to be present in 1 in 16 Inuits. Surprisingly, in spite of the lack of full-length protein in homozygous individuals, the GPV results in an attenuated form of both CMMRD in homozygous state and LS in heterozygotes. The median age of first primary cancer diagnosis in homozygotes is 22 years whereas the median age for most CMMRD and LS patients with truncating mutations is 8 years [9]. Presence of residual full-length protein in patients with homozygous *PMS2* c.2002A>G mutation is predicted to contribute to the attenuated phenotype in these patients compared to the typical CMMRD phenotype [9].

A number of mouse models of various MMR genes have been generated [10]. *Pms2-*null mice have been generated by deleting exon 2 [11]. Homozygous mutant (*Pms2*^*ko/ko*^) mice were found to be viable but showed an increase in microsatellite instability and developed lymphomas and sarcomas. These mice also exhibited higher incidence of base substitutions as well as small insertions and deletions that lead to frameshift mutations [11–14]. Although these mice did not exhibit increased intestinal tumor susceptibility, a significant increase in adenoma formation in the small intestine and colon was observed on a *Apc*^*Min*^ (a single point mutation in murine *Adenomatous polyposis coli*, *Apc*) mutant background [12, 15]. *APC* functions as a housekeeper of cellular proliferation whereas the MMR genes are considered as caretaker genes and directly inhibits the promotion of tumor growth. *APC* mutations can be detected both in familial adenomatous polyposis (FAP) and non-FAP tumors including HNPCC and sporadic colorectal cancer. It had been reported that colorectal cancers with and without defect in MMR genes involve different genes including *APC*, though defective MMR is considered to be the primary event in HNPCC [16, 17]. Endonuclease-deficient (*Pms2*^*E702K/E702K*^) knock-in mouse exhibited a higher level of genomic mutation rate and class switch recombination with increased incidences of lymphomas but failed to show an increased level of intestinal tumor formation. Unlike *Pms2*^*ko/ko*^ mice, *Pms2*^*E702K/E702K*^ male mice are fertile without any defect in spermatogenesis indicating that the endonuclease function of PMS2 is dispensable for spermatogenesis [18].

Given the high prevalence of the *PMS2* c.2002A>G variant among Inuits, generation of a mouse model that mimics this mutation would be a valuable tool in efforts to prevent the early onset cancers occurring in an under-served Canadian population. Here, we describe the generation of a knock-in mouse model of *Pms2* c.1993A>G that is the murine equivalent of the *PMS2*-founder mutation. As observed in humans homozygous for the GPV, *Pms2* transcripts in the mutant mice showed skipping of the last five bases of exon 11. This is predicted to result in premature truncation of PMS2 protein at codon I665. These mice exhibited no overt phenotype. However, on a *Apc*^*tm1Rak*^ (a chain-terminating mutation at codon 1638 in exon 15 of *Apc*) mutant background, *Pms2*^*c.1993A>G/c.1993A>G*^ mice showed a significant increase in intestinal adenoma formation, which makes them a useful model to test the efficacy of therapeutics designed to suppress aberrant splicing in heterozygotes.

## Results

### Generation of *Pms2*^*c.1993A>G/c.1993A>G*^ mice

To generate a mouse model for *PMS2* c.2002A>G mutation, we first analyzed the corresponding *Pms2* c.1993A>G mutation using the Splice Site analysis tool of Human Splicing Finder (http://www.umd.be/HSF/HSF.shtml). In silico prediction tool identifies generation of a stronger 5´splice site motif (GAG**g**ttaag) due to c.1993A>G mutation compared to the authentic splice site (TAGgtactg) [score is 91.28 for the newly generated site and 74.96 for the authentic site]. This is in agreement with the effect of the corresponding mutation in humans [9]. Analysis of the genomic sequence using ESEFinder (http://krainer01.cshl.edu/tools/ESE2/) also identifies a new binding site of the splicing factor SF2/ASF in both human and mouse genes with the mutation (Supplementary Fig. 1) [19]. Based on these observations, we predicted that *Pms2* c.1993A>G may result in a splicing defect in mice similar to the *PMS2* c.2002A>G mutation in humans.

**Fig. 1:**
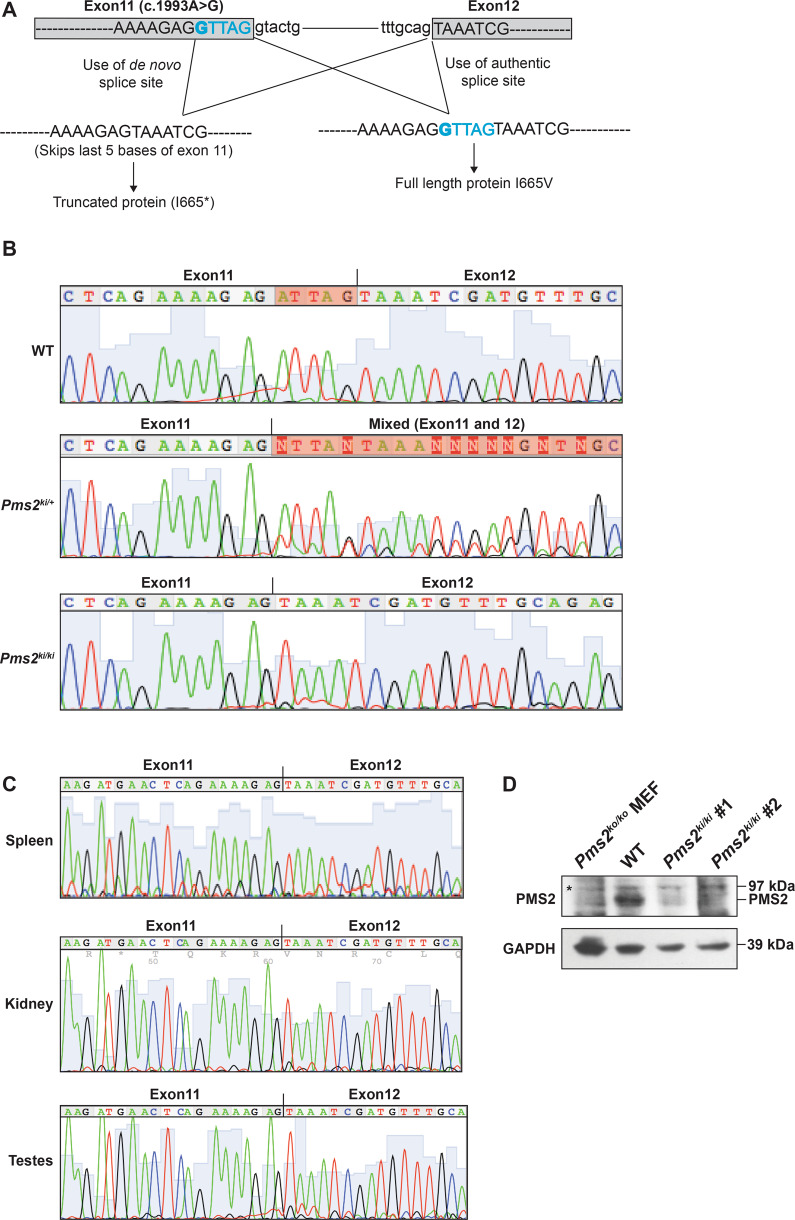
*Pms2*^*ki/ki*^ mice exhibit skipping of five bases of exon 11. **A** Schematic representation of splicing pattern due to c.1993A>G mutation. Relevant exons are shown in box and intron as straight line. Exon sequences are in capital letters and intron sequences are in small letter. The mutation is in bold and the five base of exon 11 that are deleted in the transcript due to generation of new splice site are in blue. **B** Chromatogram showing the sequence of *Pms2* transcript from colon of wild type (WT), *Pms2*^*ki/+*^ and *Pms2*^*ki/ki*^ mice. Highlighted bases in WT chromatogram shows the region skipped due to c.1993A>G mutation. Transcript sequence from heterozygous mice showing mixed sequence is highlighted. **C** *Pms2* transcript sequences from different tissues (spleen, kidney and testes) of *Pms2*^*ki/ki*^ mice showing the deletion of last five bases of exon 11. **D** Expression of PMS2 protein in wild-type (WT) and *Pms2*^*ki/ki*^ mice. Proteins were isolated from spleen of mice with indicated genotypes. Two independent *Pms2*^*ki/ki*^ mice were used and they are numbered as #1 and #2. Proteins isolated from immortalized mouse embryonic fibroblast of *Pms2*^*ko/ko*^ (null) mice (127TAg) [ATCC; cat# CRL-2817^TM^] was used as negative control. GAPDH was used as loading control. Asterisk designates non-specific band. Protein size markers are indicated on right side.

We used a conventional embryonic stem (ES)-based gene-targeting approach to introduce *Pms2* c.1993A>G into exon 11 and confirmed correct targeting by Southern hybridization and sequencing (Supplementary Fig. 2). We used targeted ES cells to generate heterozygous *Pms2*^*c.1993A*>*G-Neo/+*^ mice. We excised the *Neo* cassette by crossing these mice with *β-actin Cre* transgenic mice to obtain *Pms2*^*c.1993A>G/+*^(referred to as *Pms2*^*ki/+*^) heterozygous mice. Homozygous *Pms2*^*c.1993A>G/c.1993A>G*^ (referred to as *Pms2*^*ki/ki*^) were obtained at the expected Mendelian ratio (Supplementary Table 1). Sanger sequencing of RT-PCR products from the colon of *Pms2*^*ki/ki*^ mice showed only one transcript with a deletion of the last five base pairs of exon 11 (Fig. 1A, B). Sanger sequencing of cDNA from *Pms2*^*ki/+*^ mice showed mixed sequences in that region, indicating the use of two different splice sites in two alleles (Fig. 1A, B). The splicing defect is not tissue specific and all the tissues that were tested showed the use of the de novo splice site in *Pms2*^*ki/ki*^ mice (Fig. 1C). Deletion of five base pairs generate a stop codon at codon 665 (I665*). We also failed to detect the expression of any residual WT protein by western blot using splenic protein extract from *Pms2*^*ki/ki*^ mice (Fig. 1D). This suggests that the majority of the protein generated in these mice is truncated protein. However, we cannot rule out the possibility that very low amount of full-length protein is expressed in these mice, which is undetectable by western blotting.

### *Pms2*^*ki/ki*^ mice are fertile

**Fig. 2:**
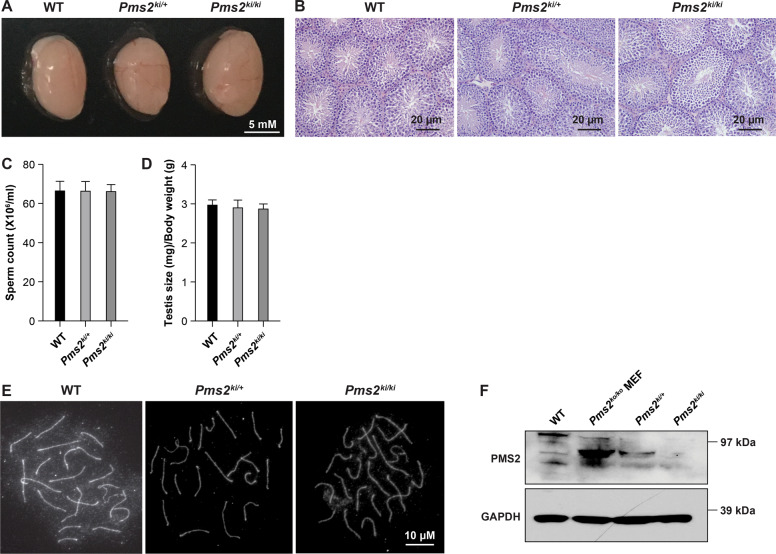
*Pms2*^*ki/ki*^ mice have normal testis morphology and histology, sperm count and meiotic progression. Testis morphology of *Pms2*^*ki/ki*^ mice: **A** Representative images of testis dissected from WT, *Pms2*^*ki/+*^ and *Pms2*^*ki/ki*^ mice. **B** H&E staining of cross sections of testis from 8 weeks old WT, *Pms2*^*ki/+*^ and *Pms2*^*ki/ki*^ mice. **C** Count of sperms isolated from epididymis of mice of different genotypes. **D** Comparison of testis sizes of 8 weeks old WT, *Pms2*^*ki/+*^ and *Pms2*^*ki/ki*^ mice. Average of five mice per genotype is shown. **E** Representative images of pachytene chromosomes stained with SYCP3 antibody from WT, *Pms2*^*ki/+*^ and *Pms2*^*ki/ki*^ mice, indicating progression through prophase I. **F** Expression of PMS2 protein in testis of *Pms2*^*ki/+*^ and *Pms2*^*ki/ki*^ mice.

*Pms2* null (*Pms2*^*ko/ko*^) male mice are infertile with reduced testis size and abnormal mature sperm cells. Defective spermatogenesis in those mice is associated with depletion of spermatogenic cells in seminiferous tubules, and impairment in progression of prophase I of meiosis due to defective synapsis [11]. Strikingly, both male and female *Pms2*^*ki/ki*^ mice are fertile and produce litters of normal size. Testis size, histology and sperm count in *Pms2* ^*ki/ki*^ mice was similar to the *Pms2*^*ki/+*^ littermates (Fig. 2A–D). Proper synapse in *Pms2*^*ki/ki*^ mice indicates that the progression through meiotic prophase I is normal in these mice (Fig. 2E). In spite of the lack of any apparent fertility problems or defects in meiotic progression in these mice, we failed to detect PMS2 protein in the testis of *Pms2*^*ki/ki*^ mice by western blotting (Fig. 2F). This suggests that the expression of functional PMS2 is highly reduced in these mice and beyond the level of detection by western blotting. However, the levels are sufficient to suppress the phenotypes observed in *Pms2 null* mice.

### Increased adenomatous polyp formation in *Pms2*^*ki/ki*^ mice

**Fig. 3:**
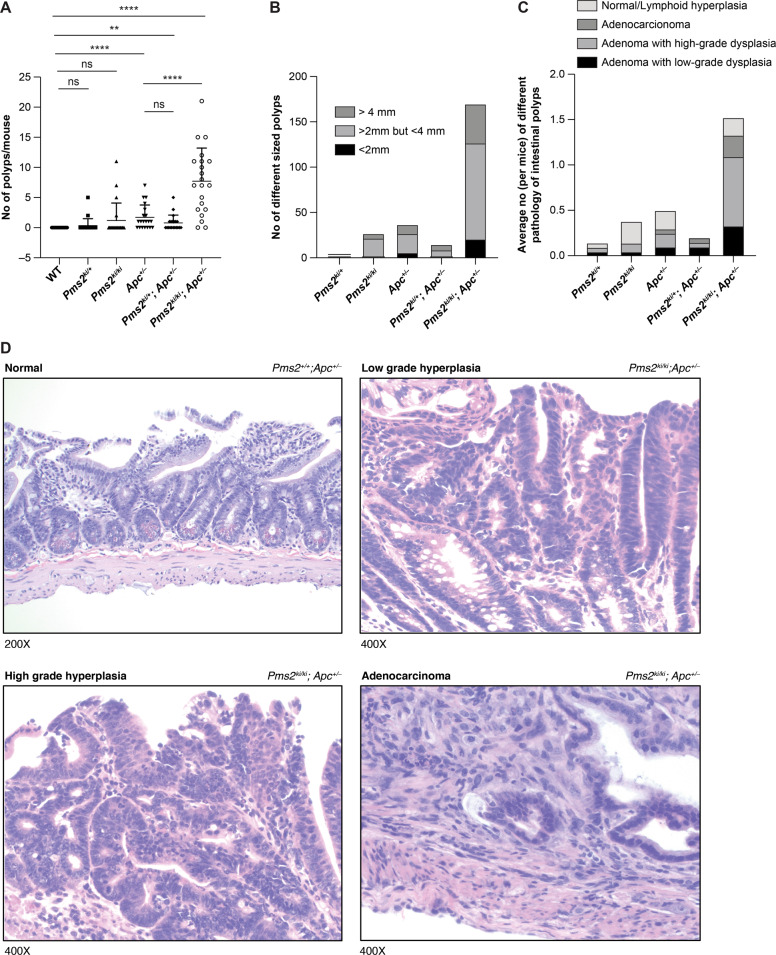
Effect of *Pms2* c.1993A>G mutation on intestinal polyp formation on a *Apc*^*+/−*^ mutant genetic background. **A** Numbers of intestinal polyps in 6 months old mice of different genotypes. For each group of genotypes 10 males and 10 females were included. Error bar shows mean ± s.d. ****P value < 0.0001 and **P value < 0.01. ns is non-significant. *P* values were calculated using Mann–Whitney test by Prism software. **B** Size distribution of intestinal polyps isolated from different genotypes. **C** Pathological distribution of intestinal polyps isolated from different genotypes. Average number was determined by dividing the total number of particular pathology found in each genotype with the number of mice studied in that group. **D** Representative images of different pathology (normal, low-grade hyperplasia, high-grade hyperplasia, and adenocarcinoma) of intestinal polyps. The genotypes are indicated on upper right corner and the magnification is indicated on lower left corner.

We next examined the intestinal tract of 6 months old mice to determine if the depletion of PMS2 protein due to aberrant splicing has any effect on intestinal polyp formation. Previous studies have revealed that PMS2 deficiency has significant effect on intestinal adenoma formation only when it was combined with APC deficiency [15]. Therefore, we crossed our *Pms2*^*ki/ki*^ mice with *Apc*^*tm1Rak/+*^ (for simplicity, referred to as *Apc*^*+/−*^) and generated *Pms2*^*ki/+*^*; Apc*^*+/−*^ and *Pms2*^*ki/ki*^*; Apc*^*+/−*^ mice [20]. *Apc*^*tm1Rak/+*^ mice display a milder cancer phenotype and live longer compared to other *Apc* mutant strains, such as *Apc*^*Min*^ and *Apc*^Δ*716*^ [20–23]. This property makes this mouse as an ideal model to study additional risk factors like MMR deficiency in intestinal tumorigenesis and has been used to study the roles of other MMR genes like *Mlh1*, *Msh2*, *Msh3*, and *Msh6* in intestinal tumorigenesis [24–26]. Intestinal polyp formation was compared at 6 months of age and 20 mice (10 males and 10 females) were examined in each group. In our facility, *Apc*^*+/−*^ mice developed 1.7 ± 2.05 (mean ± s.d) intestinal polyps whereas *Pms2*^*ki/ki*^*; Apc*^*+/−*^ mice showed increased number of polyp formation (7.7 ± 4.9) (*P* value < 0.0001) (Fig. 3A). In contrast, *Pms2*^*ki/+*^*; Apc*^*+/−*^ mice did not show significant increase in polyp formation when compared to *Apc*^*+/−*^ mice (0.8 ± 1.2 vs 1.7 ± 2.05 with *P* value 0.1246) (Fig. 3A). *Pms2* deficiency alone did not significantly increase polyp formation compared to WT mice (*P* value 0.1069) (Fig. 3A).

The polyps isolated are mostly >2 mm in size and the size distribution among different genotypes have a similar pattern (for example 149/170 are >2 mm in size for *Pms*^*ki/ki*^*; Apc*^*+/−*^ mice and 31/37 are >2 mm in size for *Apc*^*+/−*^ mice) (Fig. 3B). Polyps were further analyzed histo-pathologically (Fig. 3C, D). Average number (per mice) of intestinal polyps that were adenomatous with high-grade and low-grade dysplasia are higher (0.76 and 0.33 respectively) compared to the similar pathology observed in *Apc*^*+/−*^ (0.15 and 0.1 respectively) or *Pms2*^*ki/+*^*; Apc*^*+/−*^ (0.05 and 0.1 respectively) (Fig. 3C and Supplementary Table 2). Five polyps of *Pms2*^*ki/ki*^*; Apc*^*+/−*^ mice progressed through adenocarcinoma formation (Fig. 3C and Supplementary Table 2). We have observed similar pathology of the polyps isolated from *Apc*^*+/−*^ or *Pms2*^*ki/ki*^*; Apc*^*+/−*^ mice indicating that PMS2 deficiency due to the splicing defect significantly increased the incidence of adenomatous polyp formation when combined with APC deficiency but did not alter the morphology of the polyps themselves (Fig. 3C and Supplementary Table 2) [27].

### Microsatellite instability in adenomas isolated from *Pms2*^*ki/ki*^*; Apc*^*+/−*^ mice

**Fig. 4:**
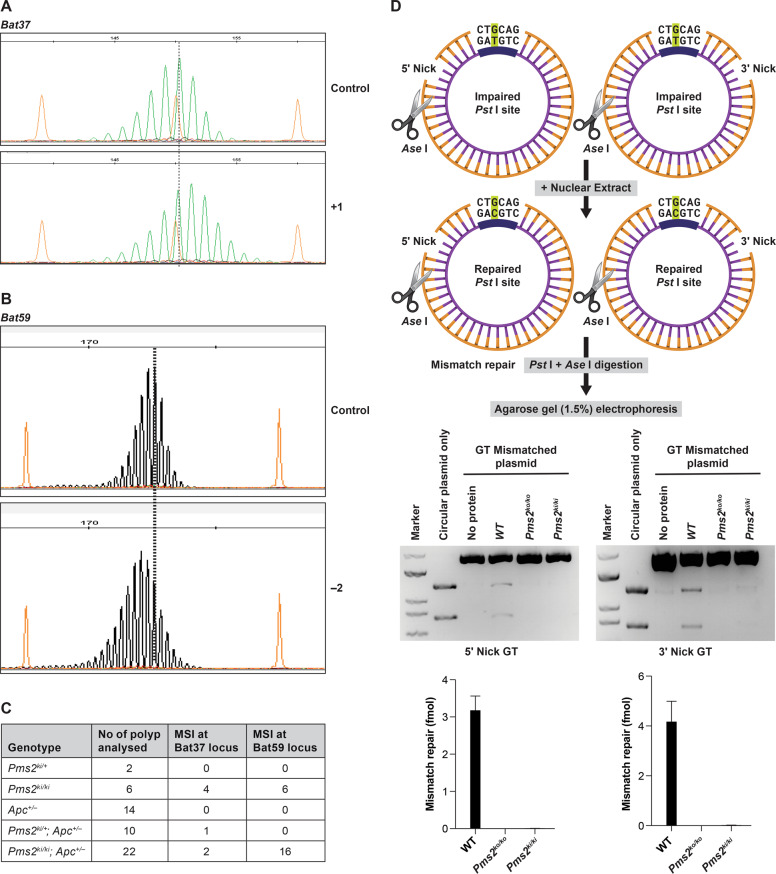
Effect of *Pms2* c.1993A>G variant on microsatellite instability (MSI) of polyps. Pattern of mononucleotide repeat markers **A** *Bat37*, **B** *Bat59* in wild-type mouse colon DNA (control) and polyp DNA of *Pms2*^*ki/ki*^ mice. Orange peaks show the size standards. Highest peak in control DNA is marked by dotted line. Number of nucleotides shifted is indicated on right. **C** Table showing the numbers of polyps with MSI in *Bat37* and *Bat59* locus of different genotypes. **D** MMR activity of nuclear extracts from MEFs of different genotypes. Top panel shows the schematic representation of the assay. *Pst* I site is impaired due to G-T mismatch in the nicked (5′ or 3′ to the mismatch) heteroduplex in which the thick line of inner strand represents unmethylated region. After incubating with nuclear extracts, heteroduplexes were digested with *Pst* I and *Ase* I. G-T mismatch repair on the *Pst* I site results into generation of smaller fragments (1.2 kb and 0.8 kb) after *Pst* I and *Ase* I digestion. Middle panel shows the agarose gel electrophoresis of digested heteroduplexes or plasmids. Lower panel shows the quantification from three independent assays.

Microsatellite instability (MSI) is a hallmark for LS. *Pms2*^*ko/ko*^ (homozygous for the null allele) mice showed MSI in the tumors, demonstrating an association between PMS2 loss and destabilized microsatellite sequences [11]. To examine MSI in the intestinal polyps obtained from our *Pms2* mutant mice, we further evaluated 54 polyps collected from mice of different genotypes (Fig. 4A). We used four mononucleotide repeat markers, *Bat24*, *Bat37*, *Bat59*, and *Bat64* for MSI analysis [28]. Among these four markers, *Bat24*, *Bat37*, and *Bat59* are monomorphic and amplified 120 bp, 149 bp, and 179 bp allele respectively (Supplementary Fig. 3). *Bat64* marker showed polymorphism and amplified three different alleles (136 bp, 162 bp, and 179 bp) in the WT mice (C57Bl6/129 mixed background) in our cohort (Supplementary Fig. 3). Due to polymorphisms observed at *Bat64* locus in our WT background, we excluded the analysis of this marker for MSI. We observed MSI consistently at *Bat37* and *Bat59* marker in the polyps analyzed (Fig. 4). MSI of polyps at *Bat37* marker revealed single repeat addition and at *Bat59* marker revealed two repeats deletion compared to WT normal colon (Fig. 4A, B). We did not observe any MSI at *Bat24* marker on the polyps analyzed compared to WT normal colon. Polyps that have showed MSI at *Bat37* and *Bat59* loci are all either *Pms2*^*ki/ki*^ or *Pms2*^*ki/ki*^*; Apc*^*+/−*^ mice except one polyp from *Pms2*^*ki/+*^*; Apc*^*+/−*^ mice that showed MSI at *Bat37* marker (Fig. 4C). Our findings suggest that the adenomatous polyps with PMS2 deficiency have increased MSI, consistent with the phenotype previously reported in *Pms2*^*ko/ko*^ mice [11].

### Endonuclease activity in primary mouse embryonic fibroblasts isolated from *Pms2*^*ki/ki*^mice

Endonuclease activity of PMS2 is important for its role in DNA repair and recombination. Mice defective in PMS2 endonuclease activity showed significant increase in genomic mutations and tumor incidence [18, 29]. To determine the effect of *Pms2 c.1993A>G* mutation on the endonuclease activity, we tested nuclear extracts isolated from *Pms2*^*ki/ki*^ and WT MEF cells for their ability to repair G-T mismatches on heteroduplexes that contains also a single-stranded nick either 5′ or 3′ to the mismatched base. Nuclear extracts from immortalized *Pms2*^*ko/ko*^ MEF (127TAg, ATCC cat # CRL-2817) was used as control. We failed to detect any MMR activity in *Pms2*^*ki/ki*^ or *Pms2*^*ko/ko*^ extracts, whereas WT nuclear extracts showed MMR activity (Fig. 4D). It demonstrates that the *Pms2*^*ki/ki*^ mice are defective in their endonuclease function and thus MMR function.

### Restoration of *PMS2* full-length transcript using Antisense Morpholino Oligos

**Fig. 5:**
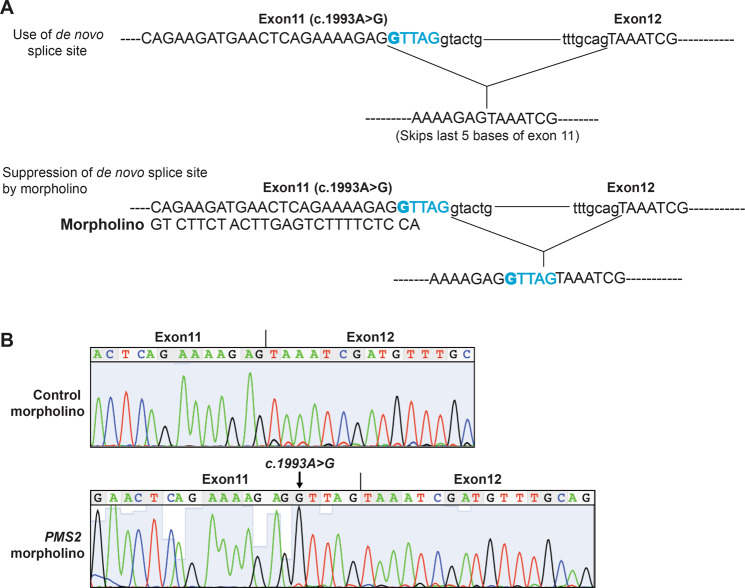
Effect of morpholino on suppression of aberrant splicing of *Pms2* c.1993A>G transcript. **A** Schematic representation of the use of morpholino to block the de novo splice site in *Pms2* transcript with c.1993A>G mutation in exon 11. Uppercase letters mark the exon sequence and lowercase letters represent intronic sequence. 5 bases that are encoded by exon 11 and deleted in the transcript due to c.1993A>G mutation are marked in blue. **B** Chromatogram showing the restoration of WT splice site in *Pms2*^*ki/ki*^ mouse embryonic fibroblast (MEF) cells. MEFs were transfected either with 10 μM control or *Pms2* specific morpholino. Arrow marks the presence of c.1993A>G mutation in *Pms2* transcript.

Antisense oligonucleotide mediated restoration of splicing defect in mutations causing Duchene Muscular Dystrophy (DMD) has entered into clinic for most common DMD mutations [30]. In order to test if use of Antisense Morpholino Oligos (AMO) can restore the splicing defect of *Pms2*^*ki/ki*^ mice, we designed morpholinos to specifically block the splicing donor site created by the mutation while leaving the authentic site, located five base pairs away, uncovered and accessible to the splicing machinery (Fig. 5A). The morpholino (10 μM) was then nucleofected into primary mouse embryonic fibroblasts derived from *Pms2*^*ki/ki*^ mouse embryos. Sanger sequencing of the RT-PCR product generated from the RNA of cells, 48 h after nucleofection of morpholinos showed that the designed morpholino could effectively block the de novo splice site generated due to *Pms2* c.1993G>A mutation (Fig. 5B). In the chromatogram, it is clear that the last five base pairs from exon 11 is included in the transcript generated after use of morpholino in the cells homozygous for *Pms2* c.1993G>A mutation (Fig. 5B, lower panel).

**Fig. 6:**
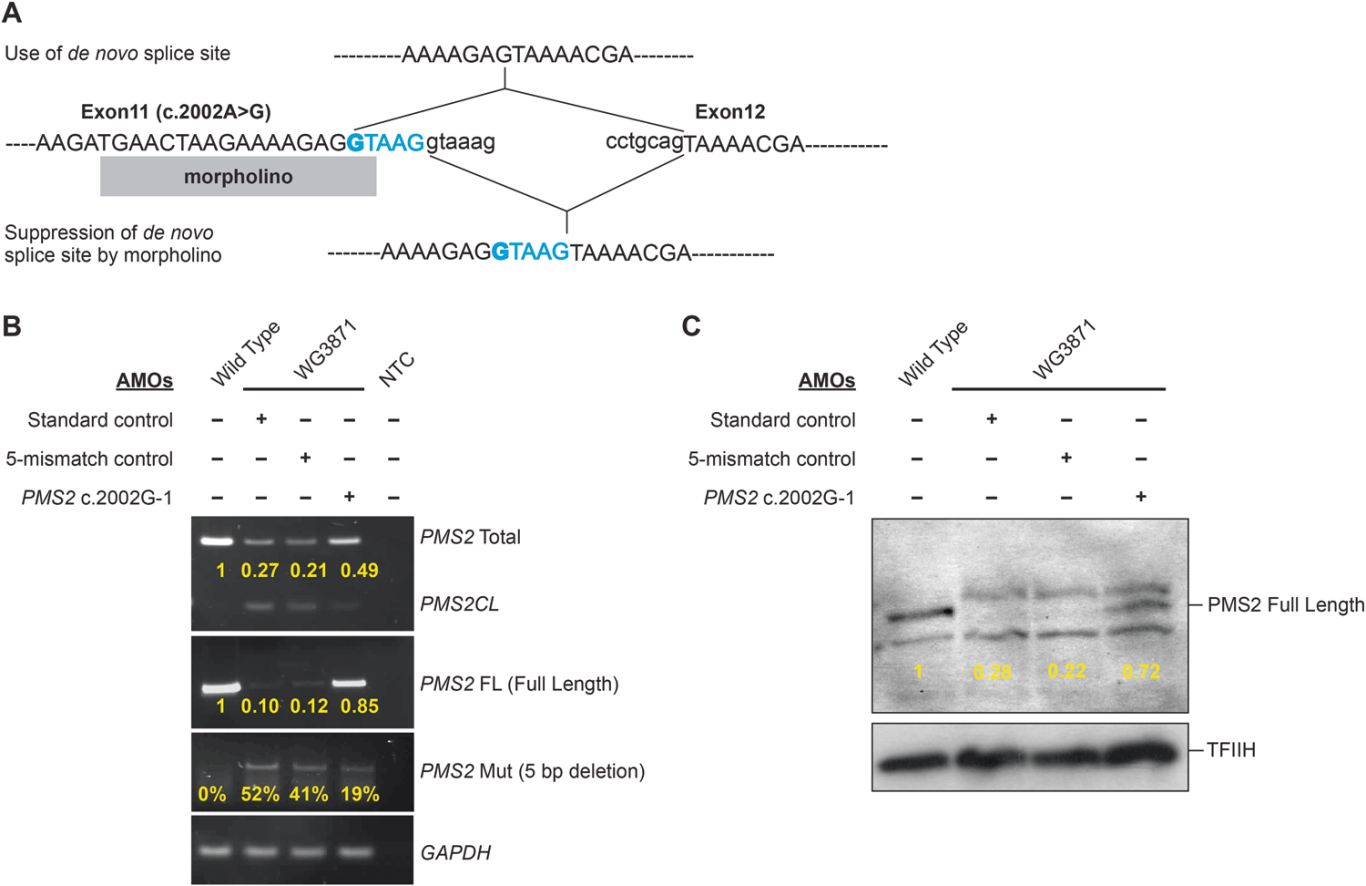
PMS2 expression in morpholino-treated human cells with *PMS2* c.2002A>G mutation. **A** Schematic representation of the splicing defect due to *PMS2* c.2002A>G mutation and use of morpholinos to block the de novo splice site. Top panel shows use of de novo splice site and bottom panel shows restoration of normal splicing by morpholinos. Morpholino is represented as a rectangular box. Exons are marked by uppercase letters and introns are in lowercase letters. 5 bases encoded by exon 11 that are deleted in the transcript due to c.2002A>G mutation are in blue and the mutation is in bold. **B** Quantitative PCR analysis of morpholino-treated cells shows an increase in the amount of full-length *PMS2* transcript in cells treated with the PMS2 c.2002-1 AMO complementary to the novel splice site compared to cells treated with mismatched and non-*PMS2* AMOs (negative controls). Treatment with the mutation-specific morpholino also results in an increase in total *PMS2* transcript (full-length and aberrantly spliced). **C** Western blot showing levels of PMS2 protein in morpholino-treated cells. There is a clear increase in full-length PMS2 in cells treated with the mutation-specific morpholino compared to barely detectable levels of PMS2 in cells treated with the controls morpholinos. TFIIH is used as a loading control. Samples: wild-type = c.2002A/c.2002A genotype; WG3871 = c.2002G/c.2002G genotype; NTC = no template control. AMOs: Standard control Morpholino specific to a region elsewhere in the genome, 5-Mismatch control Morpholino containing 5 mismatches compared to the PMS2 c.2002G-1 morpholino, PMS2 c.2002G-1 AMO morpholino complementary to the novel splice site created by the mutation.

We next tested if these AMOs can be used to restore the expression of full-length *PMS2* transcript in human fibroblasts derived from persons homozygous for the *PMS2* c.2002A>G GPV. The impact of AMO treatment on *PMS2* transcripts in treated fibroblasts was measured using quantitative PCR and gel electrophoresis as well as with pyrosequencing. Results show a marked increase in the relative and total amounts of full-length *PMS2* transcript produced in treated cells (Fig. 6A, B and Supplementary Fig. 4). In addition, western blot analysis showed a clear increase in the amount of full-length PMS2 protein produced following AMO treatment (Fig. 6C), demonstrating that treatment is effective in partially restoring normal *PMS2* expression in cells carrying the variant.

### Vivo-morpholino to correct splicing defect of *Pms2*^*ki/ki*^ mice

Morpholino oligomers attached to molecular transporters with eight guanidium head groups (Vivo-Morpholino) has been shown to correct targeted splicing errors in a wide variety of tissues including the small intestine [31]. Increased adenomatous polyp formation in the *Pms2*^*ki/ki*^ mice in combination with *Apc*^*+/−*^ genotype indicates that increasing the amount of WT *PMS2* transcript may attenuate this phenotype. Octa-guanidine linked morpholinos (vivo-morpholino) has been used previously to deliver efficiently in cardiac and skeletal muscles and improve dystrophin expression [32]. In an attempt to correct the splicing defect in *Pms2* c.1993A>G allele, we have injected *Pms2*^*ki/ki*^ mice either with control or *Pms2* specific vivo-morpholinos for 4 consecutive days. We delivered morpholinos using both intravenous (12.5 mg/kg dose) and intraperitoneal (30 mg/kg dose) injections. On day 5 post-injection, we dissected the small intestine of these mice, extracted total RNA and performed RT-PCR. Sanger sequencing of the RT-PCR products failed to detect any restoration of authentic splice site (Supplementary Fig. 5). Our results suggested that the vivo-morpholinos failed to suppress splicing from the de novo splice site. The inability to suppress aberrant splicing in the small intestine is likely due to inefficient delivery of vivo-morpholinos to the small intestine. This remains to be a challenge and must be overcome by the development of novel approaches to deliver morpholinos to these organs in future.

## Discussion

Aberrant splicing of transcripts is well known to be responsible for a number of diseases, including cancer [33, 34]. These defects arise either due to a mutation in a splicing regulator, disruption of splice sites or activation of de novo splice sites. One such splice site variant, c.2002A>G in *PMS2* is associated with LS. It is a founder GPV in one population of Canadian Inuits [9]. The *Pms2* mouse model we have generated is novel and, to the best of our knowledge, the first model with a point mutation in *Pms2* that affects splicing. Mutant mice recapitulate some of the key phenotypes observed in *PMS2* c.2002A>G homozygous individuals. In contrast to the infertility of male mice that are homozygous for the null allele (*Pms2*^*ko/ko*^), males homozygous for the *Pms2* c.1993A>G allele are fertile. *PMS2* c.2002A>G homozygous male patients are fertile and have been confirmed to have had biological children [9, 11].

The precise cause of the difference in the fertility phenotype between the two mouse models is unclear. PMS2 has three important domains: N-terminal ATPase domain, an endonuclease domain located at C-terminus, and another C-terminal domain needed for heterodimerization with MLH1. Premature truncation of the mouse PMS2 protein (I665*) due to the de novo splice site generated by c.1993A>G mutation is expected to disrupt the MLH1 heterodimerization domain whereas the ATPase domain and endonuclease domains will remain intact. It has been shown in mouse models that PMS2 endonuclease and ATPase activities are dispensable for male meiosis and fertility [18, 35].

Heterodimerization domain of PMS2 is important for male fertility and MLH1 stabilization [11, 35]. Despite the depletion of the full-length PMS2 protein to detectable levels in *Pms2*^*ki/ki*^ testis, these mice are fertile. It is possible that these mice have a low amount of the full-length protein with I665V mutation encoded by full-length transcripts generated by using the authentic exon 11 donor splice site. Our hypothesis is supported by the presence of full-length transcript in homozygous *PMS2* c.2002A>G male patients, at levels that can be detected only by highly sensitive single molecule-based Polymerase colony assay [9]. It suggests that very low levels of PMS2 is sufficient for normal meiotic progression and spermatogenesis.

PMS2 deficiency in *Pms2*^*ko/ko*^ mice was reported to increase the number of intestinal adenomas on a *Apc*^*min/+*^ background approximately three to four times relative to *Apc*^*min/+*^; *PMS2*^*ko*/ +^mice [15]. *Pms2*^*ko/+*^ mice developed more *N*-methyl-*N*-nitrosourea (MNU) induced intestinal tumors compared to WT [36]. In this study, *Pms2*^*ki/ki*^ mice in combination with heterozygous *Apc* mutation (chain-termination mutation in the 15th exon, *Apc*^*+/−*^) showed ∼4.5fold increase in intestinal polyp formation compared to *Apc*^*+/−*^ or *Pms2*^*ki/+*^; *Apc*^*+/−*^ mice. Intestinal adenomas that were *Pms2*^*ki/ki*^ also showed increased MSI, a hallmark of MMR deficiency.

Antisense oligonucleotides are among the most promising therapeutic candidates for such genetic diseases associated with splicing defects. Inducing alternate transcription by antisense oligonucleotides to bypass premature stop codon generated due to RNA mis-splicing can restore the gene function. It has been demonstrated that antisense morpholino oligonucleotides (AMO) can restore translational reading frames that were affected by splicing site mutations in mouse and dog models for Duchene muscular dystrophy (DMD) in vivo [37–40]. Morpholino based therapy for DMD has also now been approved by US Food and Drug administration as an exon-skipping drug [41]. Antisense morpholino oligonucleotides have been also used in vivo in mouse models to downregulate protein levels in splenocytes [42]. Given the high prevalence of *PMS2* c.2002A>G founder GPV and the success of ASOs in targeting aberrant splicing, we sought to explore the possibility of using morpholinos for cancer prevention associated with this mutation. The relevance of this approach was supported by our ability to suppress the de novo splice site in primary fibroblasts generated from our mouse model as well as in fibroblasts generated from patient carrying homozygous *PMS2* variant. Use of morpholinos restored expression of full-length PMS2 in these cells. However, we did not observe any effect of these vivo-morpholinos when they were administered by IP or IV injections in our mouse model. Our inability to suppress aberrant splicing in the small intestine of mice suggests that although morpholinos can be effectively delivered to some tissues in mice and patients and used to treat some diseases, efficient delivery to other tissues such as the small intestine can be challenging at present [30, 32]. Future development of innovative delivery approaches may make it feasible to target morpholinos to the small intestine. Also, development of novel CRISPR-based genome editing approaches may open new avenues for suppressing aberrant transcript splicing. Having access to our preclinical mouse model will be of immense value in testing the efficacy of new strategies.

## Methods

### Reagents

Antibodies used were PMS antibody (C-20) from Santa Cruz Biotechnology (1:1000 dilution; cat # sc-618), PMS2 from BD Biosciences, Mississauga, Canada (1:500 dilution; cat # 556415), GAPDH antibody from Abcam (1:50,000 dilution; cat # ab9485), and SYCP3 antibody from Abcam (1:500 dilution; cat # ab20244). All restriction endonucleases were purchased from New England Biolabs (Ipswich, MA, USA). Plasmid used in mismatch repair assay (pSCWO1) were obtained from Addgene (cat # 72300). All control, fluorescent tagged and vivo-morpholino oligonucleotides (25 nucleotides in length), were purchased from Gene Tools, LLC (Corvallis, OR, USA). Morpholinos were designed complementary to the mutant splice site. Non-PMS2 controls were purchased from Gene Tools, LLC (Corvallis, OR, USA). Sequences for morpholinos used in human and mouse cells are 5′ ACCTCTTTTCTTAGTTCATCTTCGG 3′ and 5′ ACCTCTTTTCTGAGTTCATCTTCTG 3′ respectively. All morpholinos except vivo-morpholinos were 3′ carboxyfluorescein labeled.

### Knock-in mouse generation

The 9.5-kb fragment containing the region from 3′part of intron 9 to 5′part of intron 14 containing *Pms2*^*c.1993A>G*^ mutation in exon 11 was used to target V6.4 (129 X C57BL/6J) embryonic stem cells to generate the knock-in allele. The positive selection gene *neo*^*r*^ flanked by two *loxP* sites inserted into intron 11 of the *Pms2* gene in the targeting construct. Two *Bam*HI sites were engineered on either side of the selection marker for screening of the targeted allele. A negative selection marker, herpes simplex thymidine kinase gene (*TK*) was also inserted into the targeting construct (Supplementary Fig. 2A). The correctly targeted ES cells were screened by Southern hybridization of *Bam*HI digested genomic DNA (Supplementary Fig. 2B, C). Presence of mutation was confirmed by sequencing of the PCR-amplified fragment (373 bp amplified using primers 5′ TAAGAGAATCGTGCTCCTCG 3′ and 5′ CCACACTGGAGTCCTATTCC 3′) surrounding the mutated region (Supplementary Fig. 2D). Resulting chimeras from these ES cells successfully transmitted the mutated allele after breeding with WT mice in the C57BL/6J × 129/Sv mixed genetic background. All animal studies were performed as per the protocols outlined in the Guide for the Care and Use of Laboratory Animals and approved by the NCI-Frederick Animal Care and Use Committee.

### RNA isolation and RT-PCR

Total RNA from frozen tissues was extracted using RNA-BEE (Tel-Test, Inc.) according to the manufacturer’s protocol. Total RNA from cell lines was isolated either using the RNeasy mini kit (Qiagen, Cat# 74104) or RNA-BEE (Tel-Test, Inc.).

RT-PCR analysis was performed using one-step RT-PCR kit (Qiagen) or by cDNA synthesis using Quantitech Reverse transcription Qiagen, kit # 205311) according to the manufacturer’s protocol. The primers from exon 11 (5′ ATCAAGTCTAGGGGTCCAGAGACTGC 3′) and exon 14 (5′ TGGAAGCAAACATCTGTCTGACTCG 3′) of *Pms2* were used for RT-PCR. The DNA amplified was gel purified (Qiagen) and sequenced. Amplified PCR products were visualized on agarose gel. Band intensity was quantified using the ImageJ software (http://rsbweb.nih.gov/ij/download.html). The primers used for RT-PCR analysis of morpholino-treated human fibroblasts are listed in Supplementary Table 3.

### Expression analysis

Frozen tissues were homogenized and proteins were extracted using the extraction buffer (50 mm Tris-HCl, pH 7.4, 1 mm ethylenediaminetetraacetic acid, 150 mm NaCl, 0.1% sodium dodecyl sulfate, 1% Triton X-100, 0.25% sodium deoxycholate, 1 mm sodium fluoride, and 1 mm orthovanadate). Proteins were separated using NuPAGE 4–12% gradient gel (Invitrogen) or 7.5% acrylamide gel using electrophoresis for western blot analysis. ECL plus western blotting detection system (Amersham) or SuperSignal West Femto kit (Thermo Scientific, Rockford, United States) were used for chemiluminescent detection.

### Selection of sample sizes

Sample sizes for animal studies were calculated using resource equation method. All analysis of animal studies results were done blindly.

### Histology

Testes and polyps were fixed in 10% neutral buffered formalin, dehydrated in ethanol, embedded in paraffin, serially sectioned, and stained with hematoxylin and eosin. Slides were examined using bright field microscopy.

### Spermatocyte spread preparation

Spermatocyte spreads from the testes of mutant and control animals were prepared and stained as described previously [43].

### Microsatellite instability analysis

PCR amplification of *Bat24*, *Bat37*, *Bat59*, and *Bat64* was carried out using Taq polymerase (Invitrogen), with primer concentrations 0.1 μM. The thermal cycling conditions were as follows: initial denaturation at 95° (Celsius) for 10 min; followed by 40 cycles of 95° for 45 s, 55° for 45 s, and 72° for 1 min; then a final extension step at 72° for 5 min. The dyes used for labeling the PCR products are FAM (*Bat24*), VIC (*Bat37*), NED (*Bat59*), and PET (*Bat64*). PCR fragments were separated by capillary electrophoresis in ABI3130XL (Life Technologies) and analyzed by using the GeneMapper program (Life Technologies). Primers used are listed in Supplementary Table 3.

### Mismatch repair assay

Nuclear extracts from MEFs were prepared as described previously [44]. Briefly cell pellets collected from 4–5 × 10^8^ cells were washed once with 40 ml of cold hypotonic buffer (20 mM Hepes, pH 7.5; 5 mM KCl; 0.5 mM MgCl_2_; 0.1% PMSF; 2 mM DTT; 1 μg/ml of each aprotinin, leupeptin, pefabloc, and E-64 (Roche)) containing 0.2 M sucrose. The cell pellet were then homozenized in 10 ml cold hypotonic buffer without sucrose. The nuclear pellet obtained after centrifugation was resuspended in 2.5 ml of extract buffer (50 mM Hepes, pH 7.5; 10% sucrose; 0.1% PMSF; 2 mM DTT; 1 μg/ml of each aprotinin, leupeptin, pefabloc, and E-64) and mixed on a rotator for 1 h after addition of 0.03 vol of 5 M NaCl. After centrifugation at 15,000×*g* for 30 min, the supernatant was concentrated with an Amicon Ultracel-10K (Millipore).

sThe preparation of 5′ G-T heteroduplex and 3′ G-T heteroduplex DNAs and in vitro MMR assays were performed as described by Geng et al. [44]. The primers used for heteroduplex generation are listed in Supplementary Table 3. In brief, MMR assays were carried out using 75 fmol (100 ng) of nicked pSCW01_GT DNA substrate, 100 μg of nuclear extract, 0.1 mM each of four dNTPs, in the standard MMR buffer (20 mM Tris-HCl, pH 7.6; 1.5 mM ATP; 1 mM glutathione; 5 mM MgCl_2_; and 110 mM NaCl, 50 mg/ml BSA) for 15 min at 37 °C. The reaction was terminated by the addition of 80 μl of stop solution (25 mM EDTA, 0.67% sodium dodecyl sulfate, and 90 μg/ml proteinase K). DNA was extracted twice with phenol/chloroform and twice with chloroform and precipitated with 2.5 volume of ethanol. DNA was then dissolved in H_2_O, and digested with 4 units each of *Pst*I and *Ase*I endonuclease and 1 μg of RNAase (Qiagen) at 37 °C for 2 h. DNA was then separated by electrophoresis on a 1.5% agarose gel. Quantification of DNA band was performed using Syngene genetools software.

## Supplementary information

Supplementary Information

Reproducibility checklist

## Data Availability

The data that support the findings of this study are available from the corresponding author upon request.
