# Supplementary material for: A novel mouse model of PMS2 founder mutation that causes mismatch repair defect due to aberrant splicing
Source: Cell Death Dis. 2021 Sep 6;12(9):838. doi: 10.1038/s41419-021-04130-8 (PMC8421400; doi:10.1038/s41419-021-04130-8)
Supplement: Supplementary file 1 — Supplementary Information [file 41419_2021_4130_MOESM1_ESM.pdf]

SUPPLEMENTARY INFORMATION

Supplementary Methods

Cell culture and transfection: Human fibroblasts wild-type for *PMS2* (Coriell #GM00321A) and homozygous for *PMS2 c.2002A>G* (WG3871) and primary mouse embryonic fibroblasts (MEF) were cultured in DMEM supplemented with 10% fetal bovine serum, 50 ug/ml Streptomycin-Penicillin at 37°C in a humid, 5% CO2 incubator. MEFs were generated from 13.5 dpc embryos. Morpholinos were delivered to the cell either using Endo-Porter delivery reagent (supplied by Gene Tools) or by nucleofection (Lonza Biosciences, USA) according to the manufacturer's protocol. Transfection efficiency was confirmed by fluorescence analysis, then morpholino-treated cells were trypsinized and washed with PBS and total RNA was isolated. All cell lines were tested for mycoplasma.

Pyrosequencing: A *PMS2*-specific, 1 kb cDNA template was amplified from RNA extracted from morpholino-treated fibroblasts using long-range PCR to serve as template for pyrosequencing. Primers used are listed in Supplementary Table 3. Optimization experiments revealed that quantitative analysis by pyrosequencing was most reliable and reproducible when transcripts were analyzed 3´ to 5´ because analysis 5´ to 3´ yielded sequence-specific artifacts. The sequencing primer was designed to overlap the exon 10-exon 11 boundary. Using this design, pyrosequencing created 2 interpretable apparent SNPs resulting from the alignment of the different transcripts that can be used to distinguish the aberrantly spliced transcript from the full-length transcript in mutant cells. Design details are provided in Supplementary Figure 6A. Replicates were performed for each treatment to ensure reproducibility of the results. The pyrosequencing reactions were performed on a Pyromark Q24 instrument by the Genome Quebec and McGill University Innovation Center (Montreal, Canada).

In-vivo delivery of morpholino: Intravenous (i.v.) or intraperitoneal (i.p.) injections of vivo-morpholinos were carried out in 10-12 week old *Pms2* $^{c.1993A>G/c.1993A>G}$ mice. The vivo-morpholino oligomers were dissolved in phosphate buffered saline and delivered to the mice by i.v. injection once daily at 12.5 mg/kg or by i.p. injection once daily at 30 mg/kg for 4 consecutive days, sacrificed on day 5 and tissues harvested for RT-PCR analysis. Three mice were injected in each group (control or *Pms2* specific vivo-morpholino).

Supplementary Figure 1. *In silico* splicing analysis of *Pms2* c.1993A>G mutation. ESEfinder score of binding motif of different splicing factors (S2F/ASF, SC35, SRp40 and SRp55) in the genomic sequences of (**A**) human (NC_000007.14: Chr7: 5986823) and (**B**) mouse (NC_000071.6: Chr5: 143926060) with substitution (bottom panel) or wild-type (top panel) as analyzed by ESEfinder (http://krainer01.cshl.edu/tools/ESE2/). Pink arrow marks the new S2F/ASF binding site (AAGAG**G**T) originated due to A>G substitution.

Supplemental Figure 01

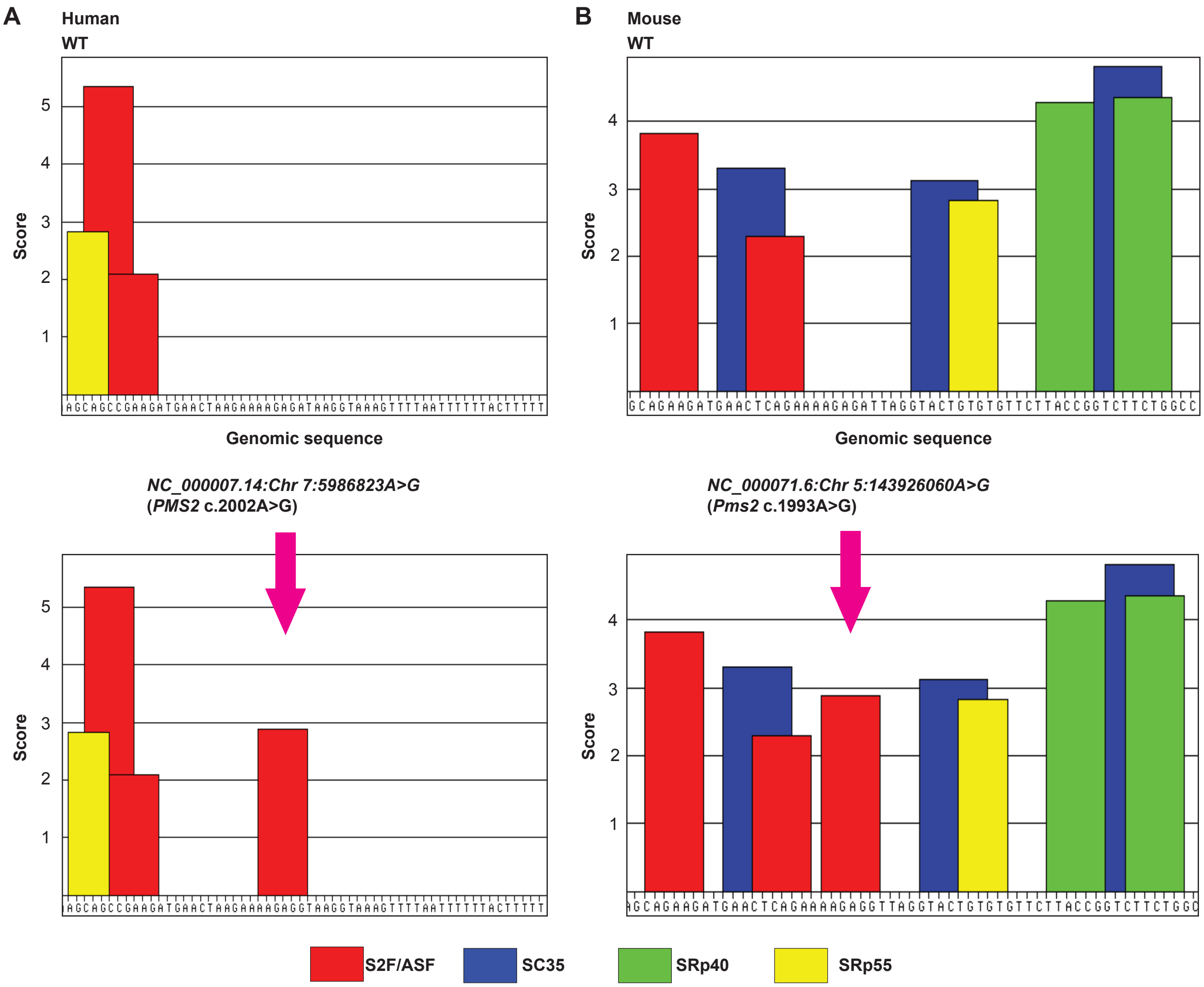

Supplementary Figure 2. Generation of *PMS2*$^{c.1993A>G}$ allele using gene-targeting. (**A**) Schematic representation of gene-targeting strategy to generate *PMS2*$^{c.1993A>G}$ allele (mutant allele). Wild type (WT) locus, targeting vector and mutant allele with *loxP-Neo-loxP* is shown. Star indicates the position of mutation. Exons are marked as filled rectangular boxes and introns are indicated as straight line. Dotted line refers to vector backbone. Position of the restriction endonuclease *Bam*HI used for screening is marked and the expected band length of WT and mutant allele is marked with dotted lines. *Thymidine kinase* (*TK*) was used as negative selection marker during gene targeting. Filled arrows indicate *loxP* sites. Thick straight lines indicate the probe used for screening. Southern blots of *Bam*HI digested genomic DNA showing the targeting of *PMS2*$^{c.1993A>G}$ allele (mutant) using probe A for 5′ region (**B**) probe B for 3′ region (**C**) of targeted locus. Size of the bands are indicated on right. (**D**) Chromatogram showing the sequence read of A>G substitution at *Pms2* heterozygous embryonic stem cells used to generate mice.

Supplemental Figure 02

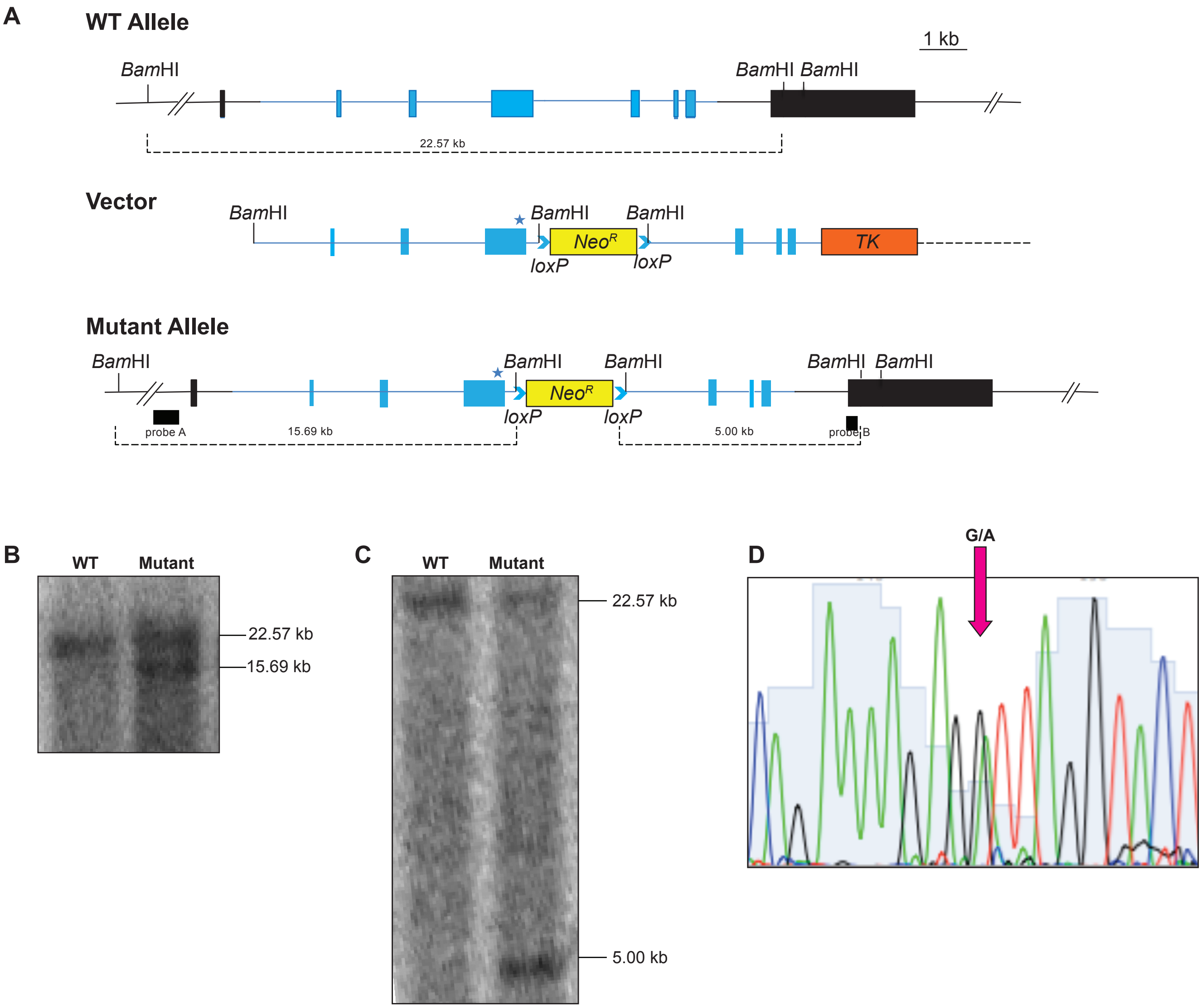

Supplementary Figure 3. Microsatellite instability (MSI) of normal colon of WT mice.

Pattern of mononucleotide repeat markers of *Bat 24* (**A**), *Bat 37* (**B**), *Bat 59* (**C**) and *Bat 64* (**D**) in two WT mice marked as #1 and #2. Highest peaks in each locus are marked in dotted line. Orange peaks in each case are from size standards. *Bat 64* locus showed two different amplification in two mice.

Supplemental Figure 03

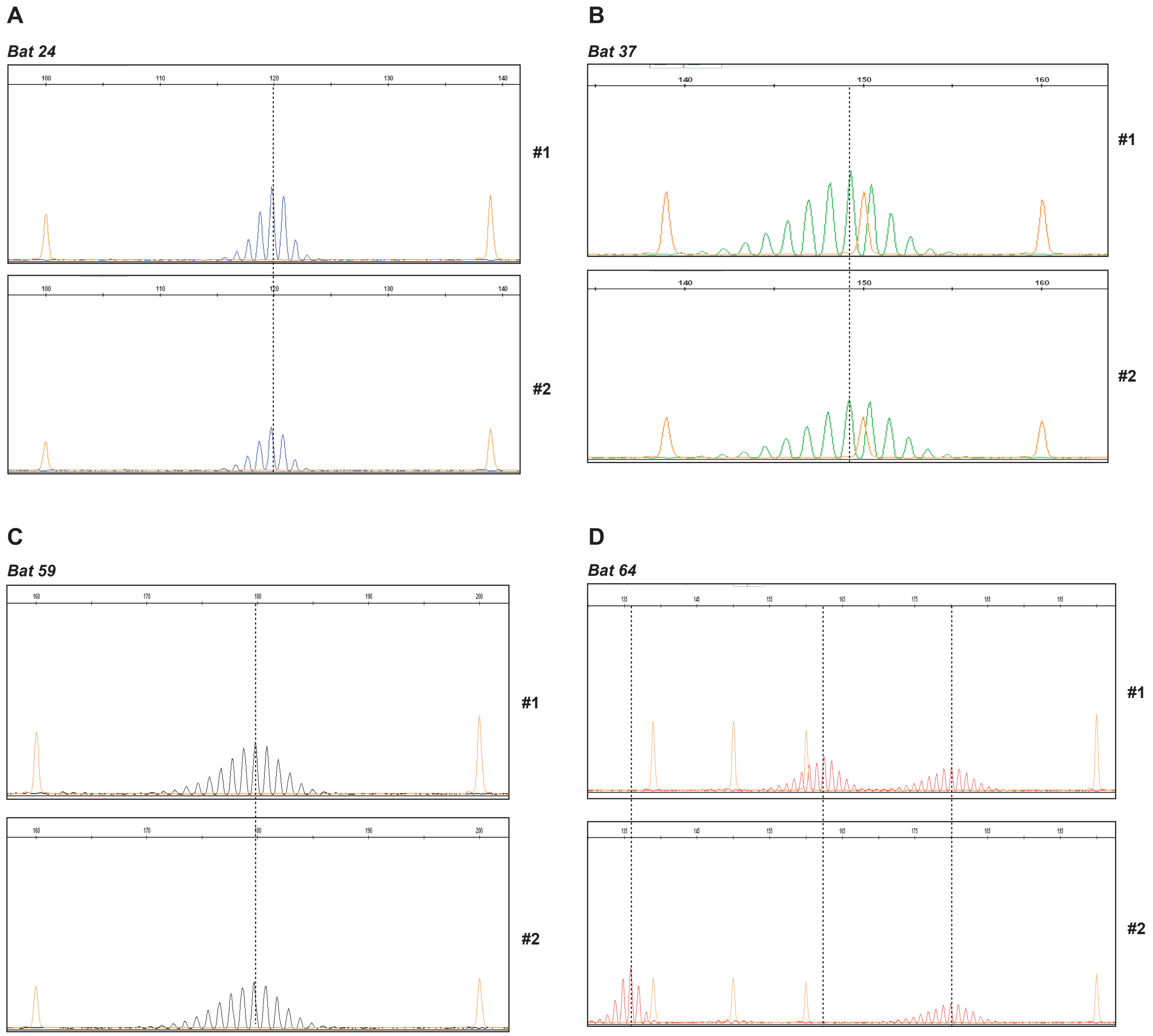

Supplementary Figure 4. Pyrosequencing analysis of PMS2 transcript after morpholino treatment in human cells with *PMS2* c.2002A>G mutation. (**A**) Pyrosequencing strategy on inverted sequence using 2 SNPs. Strategy designed to detect the apparent mismatches created during elongation when both full-length (FL) and aberrantly spliced (Mut) *PMS2* transcripts are present in homozygous mutation carriers. Specifically SNP 1 (**FL = T; Mut = C**) and SNP 2 (**FL = C; Mut = A**) are generated when both full-length transcript (properly spliced c.2002G) and mutant transcript (aberrantly spliced c.2002G containing a 5 bp deletion) begin to diverge in sequence due to the 5 bp deletion in the Mut transcript. The sequence at the top represents the order in which nucleotides will be added to the elongation mix and shows where the apparent SNPs are expected. Underlined nucleotides are non-complementary nucleotides added for baseline control. (**B**) Visual representation of the effect of AMO treatment on mutant *PMS2* transcript abundance. The relative abundance of the aberrantly spliced *PMS2* transcript containing a 5 bp deletion and the full length, normally spliced transcript was measured using pyrosequencing at two apparent SNP positions upon treatment with the control 5-Mismatches AMO, which does not successfully block splicing from the *de novo* site, and the functional AMO complementary to the 2002G mutation (PMS2 c2002G-1), which prevents splicing at the de novo site. The X axis displays the treatments, and the Y axis shows the percentage of aberrantly spliced transcript observed. Each pair of datapoints is an individual experiment. (**C**) Pyrograms generated from cell lines treated with AMOs. Panels I & II show treatment of the WG3781 cell line with the control 5-Mismatches AMO and the functional PMS2c2002G-1 AMO, respectively. Panels III & IV show replicate treatment of the WG3781 line with mismatched and functional AMOs, respectively. Panels V & VI show treatment of the cell line WG4014 with mismatched and functional AMOs, respectively. The relative percentages at the apparent SNPs of interest are calculated by the software based on peak height on each pyrogram. WG3781 = fibroblasts from patient III-3 (Family 1); WG4014 = fibroblasts from patient III-2 (Family 3).

Supplemental Figure 04

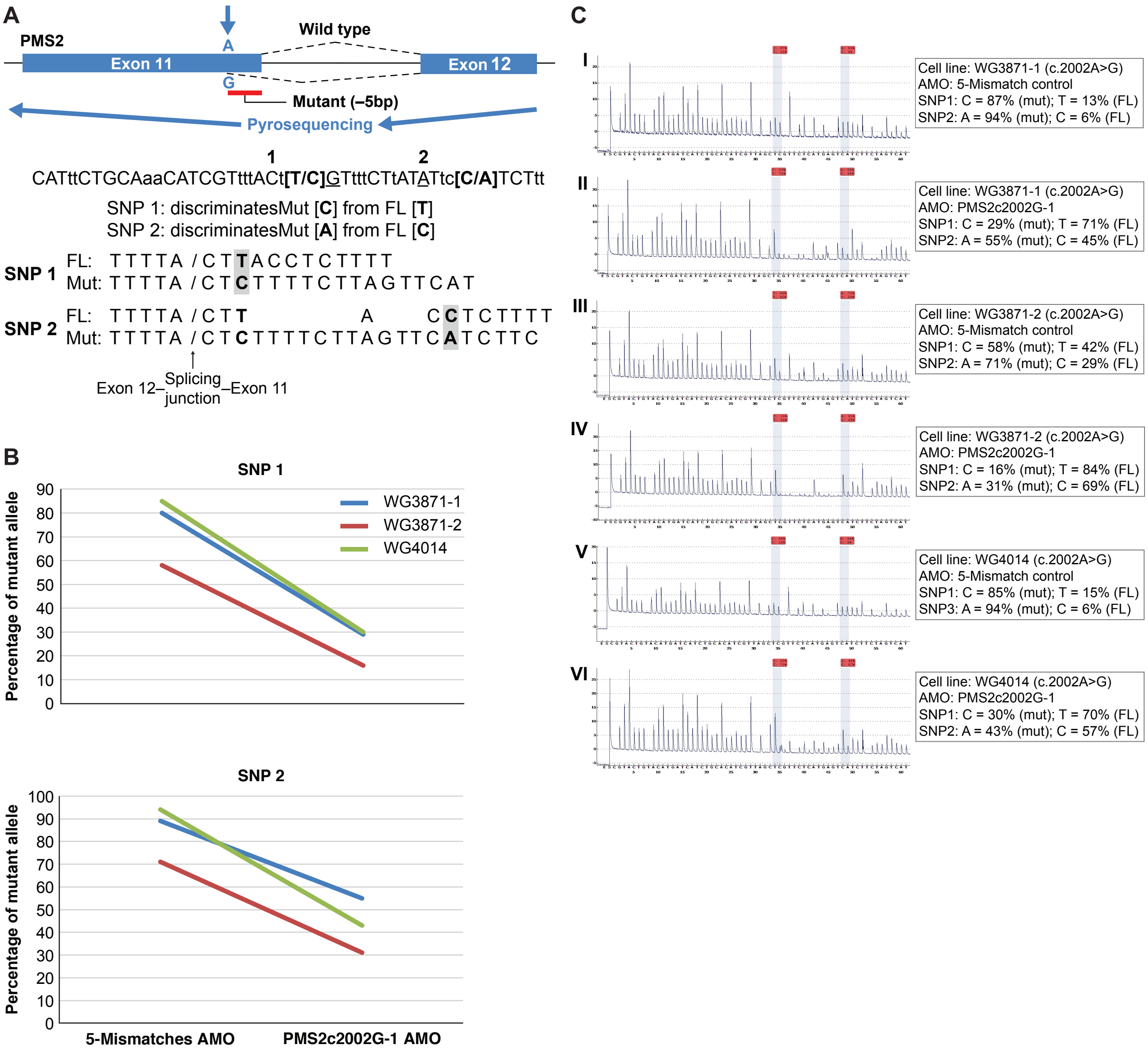

Supplementary Figure 5. Effect of injected morpholino in splicing of *Pms2*$^{c.1993A>G}$ allele.

Chromatograms of PMS2 transcript from small intestine of *Pms2*$^{ki/ki}$ mice, injected with control or PMS2 specific vivo-morpholino either (**A**) intravenously or (**B**) intraperitoneally (i.p.).

Supplemental Figure 05

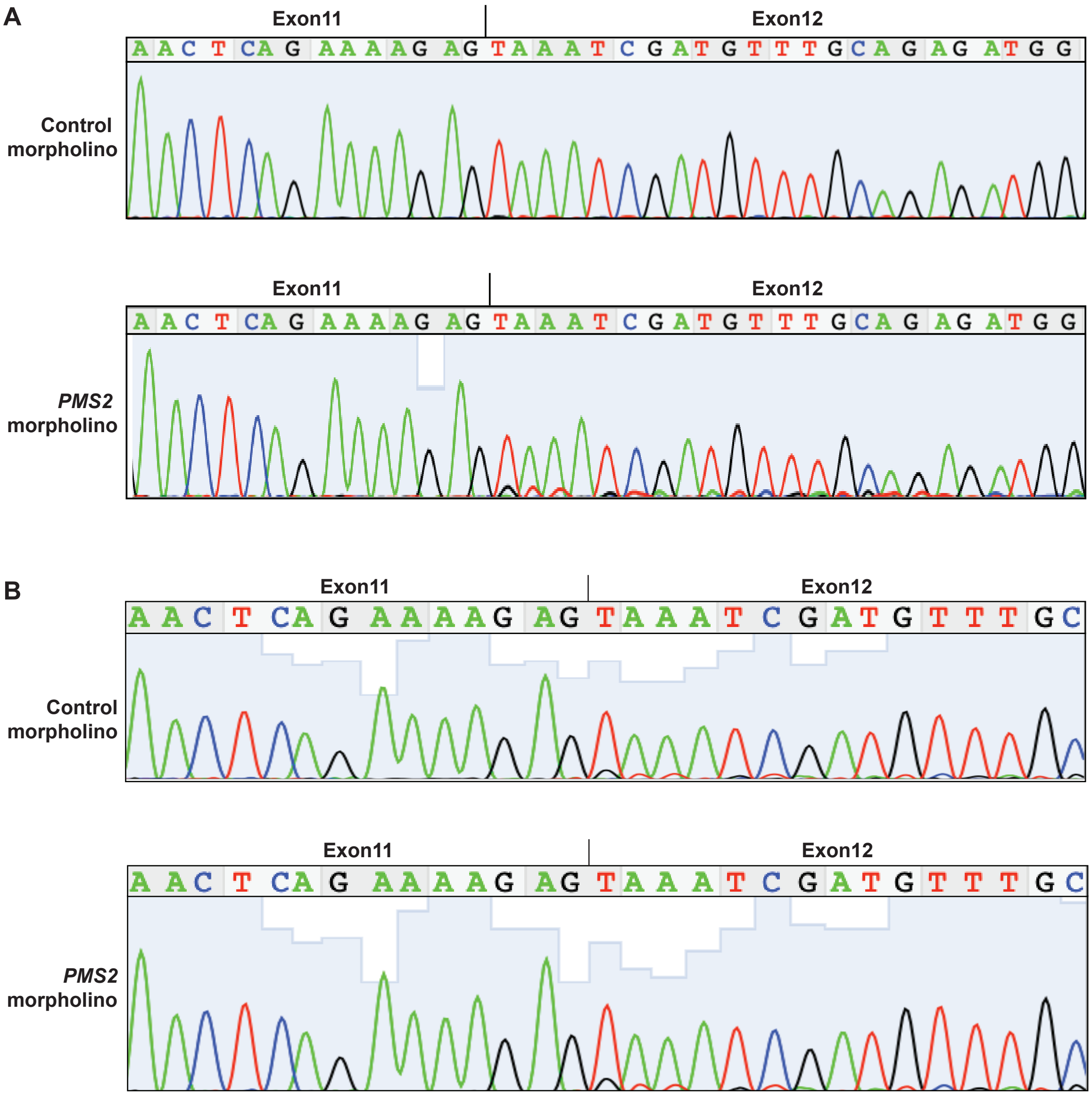

Supplementary Table 1
Observed and expected birth ratio of different genotypes obtained from *Pms2*$^{ki/ki}$ intercross.

	**WT**	***Pms2*$^{ki/+}$**	***Pms2*$^{ki/ki}$**
**Observed**	83	160	77
**Expected**	80	160	80

P value using χ^2 test is 0.9453

Supplementary Table 2

Pathology of polyps from different genotypes.

Genotype	Pathology			
	Adenoma with low grade dysplasia	Adenoma with high grade dysplasia	Adenocarcinoma	Normal/Lymphoid Hyperplasia
Pms2$^{ki/+}$	1(0.047)	1(0.047)		1(0.047)
Pms2$^{ki/ki}$	1 (0.047)	2 (0.095)		5(0.238)
Apc$^{+/-}$	2(0.1)	3(0.15)	1(0.05)	4(0.2)
Pms2$^{ki/+}$; *Apc*$^{+/-}$	2(0.1)	1(0.05)	1(0.05)	
Pms2$^{ki/ki}$ *Apc*$^{+/-}$	7 (0.33)	16(0.76)	5(0.238)	4(0.190)

Number in the parentheses shows the average number (per mice) of polyps that showed the respective pathologies.

Supplementary Table 3
Primers used in RT-PCR and pyrosequencing analysis of *PMS2*

**Primers for MSI analysis**	
Bat24-Forward	5´-CATAGACCCAGTGCTCATCTTCGT-3´
Bat24-Reverse	5´-CATTCGGTGGAAAGCTCTGA-3´
Bat37-Forward	5´-TCTGCCCAAACGTGCTTAAT- 3´
Bat37-Reverse	5´-CCTGCCTGGGCTAAAATAGA- 3´
Bat59-Forward	5´-GTAATCCCTTTATTCCATTTAGCA- 3´
Bat59-Reverse	5´-GGCTCACAACCATCCGTAACAAGA- 3´
Bat64-Forward	5´-GCCCACACTCCTGAAAACAGTCAT-3´
Bat64-Reverse	5´-CCCTGGTGTGGCAACTTTAAGC-3´
**Primers for mismatch repair assay**	
pSCWO1-1	5′- ATT TGA CTC C-3′
pSCWO1-2	5′-GAA TGA CTC GG-3′
pSCWO1-3	5′- CAT GGA CTC GCT GCA G-3′
pSCWO1-4	5′-(p)CCG AGT CAT TCC TG**T** AGC GAG TCC ATG GGA GTC AAA T-3′
**RT-PCR for *PMS2* analysis of morpholino treated cells**	
Left primer PMS2 wild-type	5´-CAAATTTTGCTACAAGAGGA-3´
Right primer PMS2 wild-type	5´-CTGCAAACATCGTTTTACTTA-3´
Left primer PMS2 mutant	5´-ATACTCAGGACATGTCAGCC-3´
Right primer PMS2 mutant	5´-CTGCAAACATCGTTTTACTCT-3´
Non-specific PMS2 forward[1]	5´-CGACACCAGTATCCATTTGTTG-3´
Non-specific PMS2 reverse[1]	5´-TACCATGGGCTTTTCCAAATCC-3´
GAPDH-Forward	5´-TGCACCACCAACTGCTTAGC-3´
GAPDH-Reverse	5´-GGCATGGACTGTGGTCATGAG-3´
**Pyrosequencing**	

Left Amplification Primer	5´-CACTGCTGGATGTTGAAGGT-3´
Right Amplification Primer[2]	5´-Biotin-GCATGCTGGTCCACTATGAA-3´
Sequencing Primer	5´-CACTGCTGGATGTTGAAGGT-3´

[1]Amplifies *PMS2* wild-type, mutant and *PMS2CL* transcripts, [2]Biotin is added to the reverse primer for anchorage to Sepharose beads.
